# Air pollution in relation to brain health indicators and global cognitive functioning in people with cardiovascular disorders along the heart-brain axis

**DOI:** 10.1016/j.cccb.2026.100535

**Published:** 2026-02-28

**Authors:** Erik J. Timmermans, Esther E. Bron, Michiel L. Bots, Anna E. Leeuwis, Justine E.F. Moonen, Frank J. Wolters, Geert Jan Biessels, Ilonca Vaartjes, Heart-Brain Connection Consortium

**Affiliations:** a Julius Center for Health Sciences and Primary Care, University Medical Center Utrecht, Utrecht University, Utrecht, The Netherlands; b Department of Radiology & Nuclear Medicine, Erasmus MC, Rotterdam, The Netherlands; c Alzheimer Center Amsterdam, Department of Neurology, Amsterdam Neuroscience, Vrije Universiteit Amsterdam, Amsterdam UMC, Amsterdam, The Netherlands; d Department of Epidemiology, Erasmus MC, University Medical Center Rotterdam, Rotterdam, The Netherlands; e Department of Neurology, UMC Utrecht Brain Center, University Medical Center Utrecht, Utrecht, The Netherlands

**Keywords:** Carotid occlusive disease, Cerebral blood flow, Heart failure, Particulate matter, Total brain volume, Vascular cognitive impairment, White matter hyperintensities

## Abstract

There is increasing evidence of an inverse relationship between air pollution and cognitive functioning. Yet, the biological mechanisms underlying this relationship remain underexplored in healthy and vulnerable populations. This study examined cross-sectional and longitudinal associations between air pollution and global cognitive functioning in individuals without or with heart failure, carotid occlusive disease, or vascular cognitive impairment. We also assessed whether the cross-sectional associations were mediated by white matter hyperintensities (WMH), total brain volume (TBV), and cerebral blood flow (CBF). The cross-sectional and longitudinal analyses included data from 341 and 180 Heart-Brain Study participants, respectively. Cognitive functioning was measured using neuropsychological tests at baseline and two-year follow-up. Brain Magnetic Resonance Imaging provided WMH and TBV in mL, and CBF in mL/100g/min. WMH and TBV were divided by total intracranial volume. Annual average outdoor concentrations of particulate matter with diameters <2.5 µm and <10.0 µm, and nitrogen dioxide in µg/m^3^ in residential six-digit postal code areas were linked to participants at baseline. Adjusted multi-level regression analyses showed no significant cross-sectional or longitudinal associations between air pollution and global cognitive functioning. The associations between air pollutants and global cognitive functioning were not significantly mediated by WMH, TBV, and CBF. In this study, air pollution was not associated with global cognitive functioning or brain health indicators in healthy individuals and those with cardiovascular disorders along the heart-brain axis. Larger studies with longer follow-up periods are warranted to extend the present findings and to further elucidate potential associations between air pollution, cognition, and the underlying biological mechanisms.

## Highlights

• We examined associations of air pollution with global cognitive functioning.
• We did this in reference participants and various heart-brain axis patient groups.
• No significant associations of PM2.5, PM10 and NO_2_ with cognition were observed.
• Associations were not mediated by MRI-derived white matter hyperintensities.
• Associations were also not mediated by total brain volume and cerebral blood flow.

## 1 Introduction

The prevalence and personal, societal, and economic burden of cognitive impairment among older adults is substantial, and is expected to rise in the next few decades due to aging populations [[1], [2], [3], [4]]. Therefore, identifying modifiable determinants of cognitive impairment in middle-aged and older adults is important in order to inform interventions that prevent or reduce cognitive impairment [[5], [6], [7]].

Environmental characteristics are increasingly recognized to affect cognitive functioning, in addition to individual-level characteristics such as lifestyle behaviours and socioeconomic position [5,6]. Air pollution is one of the environmental factors that potentially affect cognitive functioning [[8], [9], [10], [11]]. Several studies show that higher exposure to a range of largely industry- and traffic-related pollutants, such as particulate matter with diameters <2.5 µm (PM2.5) and <10.0 µm (PM10) and nitrogen dioxide (NO_2_), is associated with cognitive decline in middle-aged and older adults [[9], [10], [11]]. Higher exposure to air pollution may affect cognitive functioning through multiple, interrelated factors. These include an adverse vascular risk factor profile (e.g., obesity, hypertension, and diabetes mellitus), worse mental health (e.g., stress and depressive symptoms), and brain pathologies (e.g., neurodegeneration and vascular injury), each of which has been linked to air pollution [6,8]. Exploring, understanding, and preventing the effects of air pollution on the brain and cognitive functioning have been indicated as major global health challenges that urgently need to be addressed [12]. Yet, the associations between air pollution and brain health indicators that are potentially related to cognitive functioning remain relatively understudied [11,13].

Three examples of brain health indicators that have been related to cognitive impairment and cognitive decline in the context of ageing and vascular disease are an increased volume of white matter hyperintensities (WMH), decreased total brain volume (TBV) and reduced cerebral blood flow (CBF) [[14], [15], [16], [17], [18]]. A limited number of studies, most often focusing on middle-aged and older adults in the general population, have shown that air pollution is associated with a higher WMH burden and lower TBV and CBF [11,13,19]. The extent to which the association of air pollution with cognitive functioning is mediated by WMH, TBV, and CBF is undetermined.

The associations between air pollution in the residential environment and cognitive functioning may be more pronounced in individuals with hemodynamic disorders. Such individuals are already prone to vascular and neurodegenerative brain changes and the cognitive consequences thereof. This could make them more susceptible to the effects of air pollution [7,20].

The present study examines cross-sectional and longitudinal associations of air pollution with global cognitive functioning in individuals with or without cardiovascular disorders along the heart-brain axis, including heart failure (HF), carotid occlusive disease (COD), and vascular cognitive impairment (VCI). Furthermore, this study examines whether the associations differ across participant groups, and assesses whether the cross-sectional associations of air pollution with global cognitive functioning are mediated by WMH, TBV, and CBF. It is hypothesized that higher levels of air pollution at baseline are associated with lower levels of global cognitive functioning at baseline and at two-year follow-up. The associations are expected to be stronger in the more vulnerable patient groups compared to the relatively healthy reference group. Additionally, it is hypothesized that the inverse association of air pollution with global cognitive functioning can be partly attributed to higher levels of WMH, and lower levels of TBV and CBF.

## 2 Methods

### 2.1 Study design and study sample

Data from the Heart-Brain Study were used. The Heart-Brain Study investigates the relationships between (cardio)vascular factors, the hemodynamic status of the heart and the brain, and cognitive functioning using data from reference participants and individuals with cardiovascular disorders along the heart-brain axis (i.e., HF, COD, and possible VCI) from cardiology, memory, and neurology outpatient clinics from four sites in the Netherlands: Amsterdam UMC, location VU University medical center (AUMC-VUmc) in Amsterdam, Leiden University Medical Center (LUMC) in Leiden, Maastricht University Medical Center (MUMC) in Maastricht, and University Medical Center Utrecht (UMCU) in Utrecht [21]. The baseline data collection took place from 2014 to 2017. The two-year follow-up measurement was conducted from 2016 to 2019.

The study protocol with detailed inclusion and exclusion criteria per participant group has been described previously [21]. Most important inclusion criteria for all patient groups were a diagnosis of HF, COD or VCI according to current guidelines, age ≥50 years, ability to undergo Magnetic Resonance Imaging (MRI) and cognitive testing, and independence in daily life. People with HF were included irrespective of left ventricular ejection fraction and coronary artery disease according to the European Cardiology Society guidelines with a stable clinical situation. People with COD had a significant stenosis (i.e., >80 %) or occlusion of the internal carotid artery as assessed with Magnetic Resonance Angiography. For possible VCI, people were included with cognitive complaints (regardless of the severity of cognitive impairment (i.e., subjective cognitive decline to dementia)), combined with moderate to severe vascular brain injury on MRI, or mild vascular brain injury with presence of vascular risk factors, with a Mini-Mental State Examination score of ≥20 [22]. Most important exclusion criteria for all patient groups were clinical evidence of a brain disease other than Alzheimer Disease and VCI, a psychiatric diagnosis that affects cognitive functioning, and atrial fibrillation at the moment of inclusion. Healthy reference participants were recruited via advertisements and among spouses of people with HF, COD or VCI. The proportion of persons with cardiovascular risk factors (e.g., hypertension, hypercholesterolemia, diabetes mellitus, obesity, and currently smoking) was lower in the healthy reference group than in the patient groups.

**Fig. 1:**
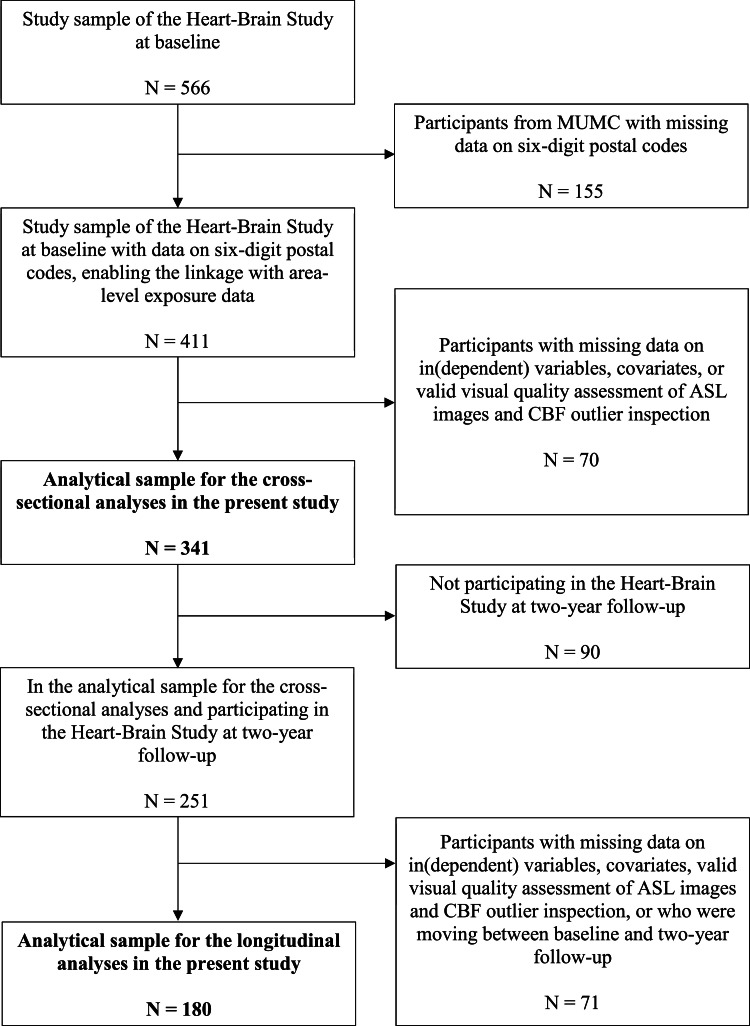
Flow diagram presenting the selection of study participants for the cross-sectional as well as longitudinal analyses^a^. Footnote: ^a^Abbreviations: ASL = Arterial Spin Labelling; CBF = Cerebral blood flow; MUMC = Maastricht University Medical Center.

Fig. 1 presents the study sample selection for the cross-sectional and longitudinal analyses. Baseline data were used in all cross-sectional analyses. The baseline sample included 566 participants (129 reference participants, 162 HF, 109 COD, and 166 VCI). Information on six-digit postal codes at baseline were lacking for all 155 participants who were included at the MUMC in Maastricht, and therefore these participants were excluded from the analyses. From the remaining sample (n = 411), participants with lacking data on the dependent variable (n = 65) and independent variables (n = 5), were excluded from the cross-sectional analyses. No participants were excluded from the analyses due to missing data on covariates or due to lacking a valid visual quality assessment of Arterial Spin Labeling (ASL) images and CBF outlier inspection. The final sample for the cross-sectional analyses consisted of 341 participants (89 reference participants, 89 HF, 78 COD, and 85 VCI) (Fig. 1).

Baseline and two-year follow-up data were used in longitudinal analyses. Of the 341 participants in the cross-sectional analyses, 251 persons completed the two-year follow-up assessment. Of them, individuals with lacking data on the dependent variable at follow-up (n = 63) were excluded from the longitudinal analyses. Furthermore, two participants were excluded due to lacking a valid visual quality assessment of ASL images and CBF outlier inspection. Additionally, six participants were excluded because they had moved between the baseline measurement and the two-year follow-up. The final sample for the longitudinal analyses consisted of 180 participants (64 reference participants, 36 HF, 43 COD, and 37 VCI) (Fig. 1).

The 161 participants who were not included in the longitudinal analyses had more often a disorder along the heart-brain axis, lower levels of global cognitive functioning and TBV, and higher levels of WMH at baseline, but they did not differ on any of the other study parameters from the included participants (n = 180).

### 2.2 Dependent variable

#### 2.2.1 Global cognitive functioning

In the Heart-Brain Study, cognitive functioning was examined using an extensive and standardized neuropsychological test battery that has been developed in the context of the Dutch Parelsnoer Initiative [23]. The various tests cover global cognitive functioning and four major cognitive domains, including: memory, language, attention-psychomotor speed, and executive functioning. For each separate cognitive domain, the various tests have been described in Supplementary File 1.

All test scores were standardized into z-scores, using reference participants as reference group. Higher z-scores implied better performance. Subsequently, the test z-scores were averaged to create four cognitive domain scores. The domain scores were based on available tests and were calculated when at least one test was available for that domain [21]. A score for global cognitive functioning was constructed by calculating the mean of the four domain scores [21,24].

### 2.3 Independent variable

#### 2.3.1 Air pollution

Based on a combination of model calculations and measurements from official measurement locations, the National Institute for Public Health and the Environment estimated the spatial variation of annual average outdoor air pollution concentrations at a resolution of 25×25 meters for various years in the Netherlands [25]. Specifically, these estimates were based on nationwide background concentration maps with a 1 km resolution, combined with local traffic information. The nationwide background concentration maps were based on dispersion models including information on industrial, vehicular, and household emissions in the Netherlands and abroad, as well as meteorological and chemical information [26]. The local vehicular traffic data were obtained from the Dutch National Air Quality Cooperation Programme [[27], [28], [29]]. Annual average outdoor air pollution concentrations, calculated from 24-hour concentration values, were estimated for official measurement locations and address locations across the Netherlands and subsequently interpolated to a nationwide raster map with a spatial resolution of 25×25 meters. Within the Geoscience and Health Cohort Consortium (GECCO), these data were further processed and made available for six-digit postal code areas, i.e., administrative areas (average area size: 0.0025 km^2^) that include, on average, 15 households [30,31]. Annual average outdoor concentrations of PM2.5, PM10, and NO_2_ in µg/m^3^ for the relevant years were linked to individuals who participated in the Heart-Brain Study at baseline, using their residential six-digit postal codes.

### 2.4 Mediators

#### 2.4.1 Three brain health indicators: WMH, TBV and CBF

The standardized brain MRI protocol that was used to assess WMH, TBV and CBF in participants of the Heart-Brain Study has been described in detail elsewhere [24]. The MRI scans were acquired on Philips Ingenia 3T scanners at LUMC and UMCU, and on a Philips Gemini 3T PET-MR scanner at AUMC-VUmc (Philips, Best, the Netherlands) [21]. For each participant, infarcts and other pathologies, that potentially affect automatic tissue segmentation, were annotated by a neuroradiologist. Subsequently, the annotated infarcts and pathologies were manually segmented by trained students [24]. A brain tissue and WMH segmentation method (Quantib B.V., Rotterdam, the Netherlands) was applied to T1-weighted and fluid attenuation inversion recovery scans [32]. Using these manual infarct segmentations, corrected volumes in mL of WMH, gray matter, normal appearing white matter, and infarcts were computed. TBV in mL was calculated by summing these volumes. To correct for head size, WMH was divided by total intracranial volume (ICV). Similarly, TBV was expressed as brain parenchymal fraction, which represents the proportion of the total ICV that is occupied by brain tissue [33]. Total ICV in mL was calculated by summing corrected volumes of WMH, gray matter, normal appearing white matter, cerebrospinal fluid, and infarcts including tissue loss. These corrected volumes were obtained using manual infarct segmentations. Other pathologies and incidental findings were not part of the total ICV [21,24,32].

Whole brain partial volume corrected cortical CBF in mL/100 g/min was examined with pseudo-continuous Arterial Spin Labelling (pCASL) (multi-slice two-dimensional Echo Planar Imaging (EPI) acquisition with background suppression; labelling duration = 1800 milliseconds; post-labelling delay = 1800 milliseconds; single show EPI readout; resolution = 3×3×7 millimeter) [24,34]. The pCASL data were processed using the automated Iris pipeline for CBF quantification, including a region-of-interest segmentation method and an ASL quantification method [24,35,36]. Quantification of ASL data into CBF maps was based on a single-compartment model after the subtraction of labelled images from control images according to the recommended approach [34]. To scale the signal intensities of the subtracted ASL images to absolute CBF units, a separately acquired proton density weighted image (M0) was used. The quantification further included motion correction of the raw ASL data and partial volume correction [24,37,38]. CBF was quantified in normal-appearing gray matter (NAGM) only. To obtain the NAGM mask for each participant, binary gray matter segmentation (Statistical Parametric Mapping, London, United Kingdom) was combined with the WMH and infarct mask [24,39].

### 2.5 Covariates

The cross-sectional analyses were adjusted for the following covariates at baseline: age in years, sex (man (reference category) versus woman), educational level, smoking status, weight status, area-level socioeconomic status and population density. All longitudinal analyses were additionally adjusted for global cognitive functioning at baseline.

Educational level was categorized into: low (Verhage categories 1–4; reference category), intermediate (Verhage category 5), and high (Verhage categories 6–7) [40,41].

Smoking status was categorized into: never smokers (reference category), former smokers, and current smokers.

Weight status was assessed using Body Mass Index, which was calculated as weight in kilograms divided by height in meters squared [42]. Objectively measured area-level socioeconomic status scores were obtained from the Netherlands Institute for Social Research [43]. These scores are based on the average income, the percentage of residents with a low income, the percentage of residents with a low level of education, and the percentage of unemployed residents in the neighbourhood. Higher scores indicate a higher area-level socioeconomic status.

Population density (i.e., number of residents/hectare) z-scores were calculated using data from Statistics Netherlands, and obtained from GECCO [30,31,44]. They were calculated for 250-meter Euclidean buffer zones around the centroid of the residential six-digit postal code area of participants in the relevant years.

### 2.6 Statistical analyses

Characteristics of the study samples and the area-level exposure measures are presented for the full samples and for each participant group, separately. One-way Analyses of Variance and Kruskall-Wallis tests, both including post-hoc Bonferroni corrections, and Pearson Chi-square tests were conducted to compare groups when appropriate.

Multi-level regression models with center (i.e., AUMC-VUmc, LUMC, and UMCU) as a second level were applied to examine the cross-sectional associations of individual air pollutants (i.e., one-pollutant models) with global cognitive functioning at baseline. Similar analyses were conducted to assess the longitudinal associations of air pollution at baseline with global cognitive functioning at two-year follow-up. The cross-sectional analyses were adjusted for age, sex, educational level, smoking status, weight status, area-level socioeconomic status and population density. The longitudinal associations were additionally adjusted for global cognitive functioning at baseline. By conducting multilevel analyses, the clustering of observations (level-1 unit) within the same center (level-2 unit) has been taken into account.

**Fig. 2:**
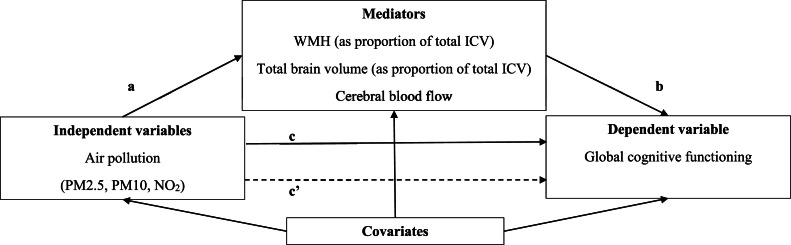
Mediation analyses conducted in this study^a,b^. Footnotes: ^a^Abbreviations: ICV = Intracranial volume; NO_2_ = Nitrogen dioxide; PM2.5 = Particulate matter with diameter <2.5 µm; PM10= Particulate matter with diameter <10.0 µm; WMH = White matter hyperintensities. ^b^The c-path represents the total effect of the relevant area-level exposure measure on global cognitive functioning at baseline. The c’-path represents the direct effect of the relevant area-level exposure measure on global cognitive functioning, after adjustment for the mediator. The ab-path represents the indirect effect. The indirect effect is estimated as the multiplication of the effect of the relevant area-level exposure measure on the mediator (i.e., a-path) and the effect of the mediator on global cognitive functioning (i.e., b-path), after adjustment for the area-level exposure measure. All associations in the mediation analyses were also adjusted for all other included covariates at baseline (i.e., age, sex, educational level, smoking status, weight status, area-level socioeconomic status and population density).

To assess the mediation effects of the three brain health indicators, the total effect of each air pollutant on global cognitive functioning was decomposed into a direct and indirect effect. A mediation effect was considered to be present when a statistically significant indirect effect was observed [45]. A sequence of standardized regression analyses was used to calculate the total, direct, and indirect effects. The total effect is the effect of the relevant area-level exposure measure on global cognitive functioning (Fig. 2; c-path). The direct effect is the effect of the relevant area-level exposure measure on global cognitive functioning, after adjustment for the mediator (Fig. 2; c’-path). The indirect effect is estimated as the multiplication of the effect of the relevant area-level exposure measure on the mediator (Fig. 2; a-path) and the effect of the mediator on global cognitive functioning (Fig. 2; b-path) after adjustment for the exposure measure [46]. In other words, the indirect effect (i.e., ab-path) quantifies the effect of the area-level exposure measure on the outcome measure that is channeled through the potential mediator [47,48]. Mediation analyses were conducted in the full study sample at baseline, and were performed irrespective of the presence of a statistically significant total effect, as the absence of a statistically significant total effect does not necessarily imply the absence of a direct and indirect effect [49]. All associations in the mediation analyses were also adjusted for all included covariates at baseline (Fig. 2). Since indirect effects usually have a skewed distribution, bootstrapping, based on 5000 bootstrap samples, was applied to estimate 95 % CIs for the indirect effects [50].

To examine whether the associations were particularly driven by one of the participant groups, the cross-sectional, longitudinal, and mediation analyses were conducted separately in each participant group. To study possible effect modification by participant group (i.e., reference participants (reference group), HF, COD, and VCI), an interaction term between each air pollution exposure measure and participant group was created. Each interaction term, together with its two main terms, were statistically tested in a fully adjusted model.

Information on whether the included participants from AUMC-VUmc moved houses between baseline and two-year follow-up was missing. A very small proportion of participants from LUMC (6.3 %) and UMCU (2.9 %) did relocate between baseline and two-year follow-up. Therefore, it was assumed that all participants at AUMC-VUmc (n = 111) did not move during this time frame, and these participants were included in the main longitudinal analyses [7]. To examine whether this assumption affected the results, the main longitudinal analyses were repeated in sensitivity analyses in which these participants were excluded.

Furthermore, additional analyses were conducted to examine the cross-sectional and longitudinal associations between air pollution and each cognitive domain separately (i.e., memory, language, attention-psychomotor speed, and executive functioning). Given the potential interactions between different air pollutants, additional analyses were also conducted to examine the cross-sectional and longitudinal associations between air pollution and global cognitive functioning using multi-pollutant models.

In all statistical analyses, a p-value below 0.05 was considered as statistically significant. All statistical analyses were performed in IBM SPSS Statistics (Version 27.0) [51]. The mediation analyses were conducted using model 4 (i.e., simple mediation model) in Hayes PROCESS v3.5.3 macro [52].

## 3 Results

Characteristics of the study sample for the cross-sectional and longitudinal analyses, and all relevant area-level exposure measures are presented in Table 1.

**Table 1:** Baseline characteristics of the study samples and area-level exposure measures.a.

Variables	Study sample included in cross-sectional analyses					Study sample included in longitudinal analyses				
	Total (n = 341)	Reference participants (n = 89)	HF (n = 89)	COD (n = 78)	VCI (n = 85)	Total (n = 180)	Reference participants (n = 64)	HF (n = 36)	COD (n = 43)	VCI (n = 37)
**Dependent variable**^b^										
Global cognitive functioning (Mean ± SD)	−0.4 ± 0.9	Ref	−0.2 ± 0.6	−0.5 ± 0.7	−1.0 ± 1.2 d^,^^e^^,^^f^	−0.3 ± 0.8	Ref	−0.3 ± 0.7	−0.6 ± 1.1 d	−0.6 (0.8) d
**Independent variables**^c^										
Annual average outdoor concentration of PM2.5 in µg/m^3^ (Mean ± SD)	11.4 ± 1.1	11.3 ± 0.9	11.5 ± 1.3	11.2 ± 1.10 e	11.4 ± 1.2	11.4 ± 1.2	11.2 ± 0.8	12.0 ± 1.5 d	11.2 ± 1.0 e	11.3 ± 1.3
Annual average outdoor concentration of PM10 in µg/m^3^ (Mean ± SD)	18.9 ± 1.4	19.0 ± 1.2	19.2 ± 1.4	18.6 ± 1.3	18.8 ± 1.5	19.0 ± 1.4	19.0 ± 1.1	19.7 ± 1.6	19.0 ± 1.2 e	18.7 ± 1.7 e
Annual average outdoor concentration of NO_2_ in µg/m^3^ (Mean ± SD)	20.8 ± 4.4	21.5 ± 4.2	22.0 ± 3.9	19.3 ± 4.0 d^,^^e^	20.3 ± 5.0 e	21.0 ± 4.2	21.5 ± 3.9	22.3 ± 4.0	19.0 ± 3.6 d^,^^e^	20.4 ± 4.8
**Mediators**										
Volume of white matter hyperintensities (as proportion of total intracranial volume) (Median (IQR))	0.001 (0.004)	0.001 (0.001)	0.001 (0.003) d	0.001 (0.001) d	0.007 (0.016) d^,^^e^^,^^f^	0.001 (0.004)	0.001 (0.001)	0.002 (0.003) d	0.001 (0.003)	0.007 (0.010) d^,^^e^^,^^f^
Total brain volume (as proportion of total intracranial volume) (Mean ± SD)	0.782 ± 0.035	0.799 ± 0.026	0.784 ± 0.036 d	0.769 ± 0.032 d^,^^e^	0.776 ± 0.037 d	0.780 ± 0.036	0.794 ± 0.028	0.782 ± 0.035	0.761 ± 0.037^d^	0.777 ± 0.039
Whole-brain partial volume corrected cortical cerebral blood flow in mL/100 *g*/min (Mean ± SD)	52.2 ± 11.7	56.3 ± 11.4	53.5 ± 11.7	47.6 ± 10.1 d^,^^e^	50.8 ± 11.8 d	52.7 ± 11.0	57.1 ± 10.6	54.3 ± 11.0	47.4 ± 9.9 d^,^^e^	49.5 ± 9.2 d
**Covariates**										
Age in years (Mean ± SD)	67.7 ± 8.6	65.6 ± 7.3	69.4 ± 10.1 d	65.2 ± 7.7 e	70.2 ± 8.1 d^,^^f^	66.9 ± 8.0	66.5 ± 6.8	68.4 ± 10.5	65.2 ± 7.9	68.0 ± 7.2
Sex (%) Women	34.9	47.2	d 32.6	d 24.4	34.1	33.9	42.2	30.6	d 20.9	37.8
Educational level (%) Low Intermediate High	23.2 32.2 44.6	20.2 29.2 50.6	28.1 30.3 41.6	d 24.4 43.6 32.1	f 20.0 27.1 52.9	23.9 32.8 43.3	17.2 32.8 50.0	27.8 30.6 41.7	25.6 39.5 34.9	29.7 27.0 43.3
Smoking status (%) Never smokers Former smokers Current smokers	27.0 57.5 15.5	46.1 46.1 7.8	d 29.2 56.2 14.6	d^,^^e^ 5.2 67.9 26.9	d^,^^f^ 24.7 61.2 14.1	30.0 57.2 12.8	48.4 46.9 4.7	33.3 52.8 13.9	d^,^^e^ 4.7 74.4 20.9	d^,^^f^ 24.3 59.5 16.2
Weight status (BMI in kg/m^2^)	26.6 ± 3.9	26.2 ± 3.9	26.8 ± 4.1	27.6 ± 3.8	25.8 ± 3.7 f	26.6 ± 3.8	26.3 ± 3.8	26.5 ± 3.9	27.8 ± 4.1	25.9 ± 3.2
Area-level socioeconomic status score (Mean ± SD)	0.4 ± 0.9	0.4 ± 0.8	0.5 ± 0.9	0.2 ± 0.9	0.5 ± 1.0	0.4 ± 0.9	0.4 ± 0.8	0.3 ± 1.1	0.2 ± 0.9	0.6 ± 0.9
Population density [z-score] (Median (IQR))	5.3 (3.9)	5.8 (4.1)	6.6 (4.5)	4.2 (4.1) d^,^^e^	4.8 (4.5) e	5.3 (3.9)	5.8 (3.4)	7.0 (5.0)	4.2 (4.4) e	4.4 (4.8)
**Other variable**										
Center (%) AUMC-VUmc LUMC UMCU	40.2 23.1 36.7	23.6 48.3 28.1	d 67.4 32.6 0.0	d^,^^e^ 0.0 9.0 91.0	d^,^^e^^,^^f^ 65.9 0.0 34.1	43.3 26.1 30.6	28.1 53.1 18.8	d 80.6 19.4 0.0	d^,^^e^ 0.0 14.0 86.0	d^,^^e^^,^^f^ 83.8 0.0 16.2

a Abbreviations: AUMC-VUmc = Amsterdam UMC, location VU University medical center, BMI = Body Mass Index, COD = People with carotid occlusive disease; g = Gram; HF = People with heart failure; IQR = interquartile range; kg = Kilogram, LUMC = Leiden University Medical Center; m = Meter; mL = Milliliter; min = Minute; n = Number; NO_2_ = Nitrogen dioxide; PM2.5 = Particulate matter with diameter <2.5 µm; PM10 = Particulate matter with diameter <10.0 µm; Ref = reference group; SD = Standard deviation; UMCU = University Medical Center Utrecht; VCI = People with possible vascular cognitive impairment.

b For the study sample included in the longitudinal analyses, the presented dependent variable has been measured at two-year follow-up.

c The air pollution data were related to the six-digit postal code areas where participants were living.

d p-value < 0.05 compared to reference participants.

e p-value < 0.05 compared to people with heart failure.

f p-value < 0.05 compared to people with carotid occlusive disease.

### 3.1 Cross-sectional associations at baseline

The cross-sectional analyses revealed no significant associations of air pollution with global cognitive functioning in the full sample (Table 2; β_PM2.5_ = 0.012, 95 % CI = -0.078 to 0.101; β_PM10_ = 0.031, 95 % CI = -0.046 to 0.108; β_NO2_ = 0.022, 95 % CI = -0.003 to 0.047). The air pollution by participant group interaction terms generally indicated that the cross-sectional associations did not differ by participant group (Table 2 and Supplementary File 2, Table 1.1).

**Table 2:** Cross-sectional associations of air pollution with global cognitive functioning at baseline. a^,^^b^^,^^c^^,^d.

Variables	Total (n = 341)	Reference participants (n = 89)	HF (n = 89)	COD (n = 78)	VCI (n = 85)
	β (95 % CI)	β (95 % CI)	β (95 % CI)	β (95 % CI)	β (95 % CI)
**Annual average outdoor concentration of PM2.5**					
Global cognitive functioning	0.012 (−0.078 to 0.101)	−0.021 (−0.137 to 0.094)	−0.055 (−0.156 to 0.046)	−0.052 (−0.187 to 0.084)	0.190 (−0.068 to 0.448)
**Annual average outdoor concentration of PM10**					
Global cognitive functioning	0.031 (−0.046 to 0.108)	0.008 (−0.088 to 0.104)	−0.041 (−0.137 to 0.054)	−0.048 (−0.163 to 0.068)	0.133 (−0.089 to 0.355)
**Annual average outdoor concentration of NO_2_**					
Global cognitive functioning	0.022 (−0.003 to 0.047)	0.003 (−0.024 to 0.029)	−0.005 (−0.039 to 0.028)	−0.018 (−0.060 to 0.025)	0.049 (−0.025 to 0.123)

a Abbreviations: CI = Confidence interval; COD = People with carotid occlusive disease; HF = People with heart failure; n = number; NO_2_ = Nitrogen dioxide; PM2.5 = Particulate matter with diameter <2.5 µm; PM10 = Particulate matter with diameter <10.0 µm; VCI = People with possible vascular cognitive impairment.

b These cross-sectional associations are adjusted for age, sex, educational level, smoking status, weight status, area-level socioeconomic status and population density at baseline.

c The air pollution data were related to the six-digit postal code areas where participants were living.

d None of the presented associations was statistically significant (i.e., p-value<0.05).

### 3.2 Longitudinal associations

None of the air pollution exposure measures at baseline were significantly associated with global cognitive functioning at two-year follow-up in the full sample (Table 3; β_PM2.5_ = 0.038, 95 % CI = -0.032 to 0.109; β_PM10_ = 0.014, 95 % CI = -0.048 to 0.076; β_NO2_ = -0.001, 95 % CI = -0.022 to 0.022). The air pollution by participant group interaction terms indicated that the longitudinal associations did not differ by participant group (Table 3 and Supplementary File 2, Table 1.2).

**Table 3:** Longitudinal associations of air pollution at baseline with global cognitive functioning at two-year follow-up.^a^^,^^b^^,^^c^^,^d.

Variables	Total (n = 180)	Reference participants (n = 64)	HF (n = 36)	COD (n = 43)	VCI (n = 37)
	β (95 % CI)	β (95 % CI)	β (95 % CI)	β (95 % CI)	β (95 % CI)
**Annual average outdoor concentration of PM2.5**					
Global cognitive functioning	0.038 (−0.032 to 0.109)	0.014 (−0.078 to 0.106)	0.057 (−0.094 to 0.208)	0.064 (−0.202 to 0.329)	−0.056 (−0.196 to 0.085)
**Annual average outdoor concentration of PM10**					
Global cognitive functioning	0.014 (−0.048 to 0.076)	0.016 (−0.057 to 0.091)	0.091 (−0.042 to 0.224)	−0.051 (−0.286 to 0.185)	−0.048 (−0.167 to 0.070)
**Annual average outdoor concentration of NO_2_**					
Global cognitive functioning	−0.001 (−0.022 to 0.022)	0.008 (−0.011 to 0.026)	−0.021 (−0.065 to 0.024)	0.013 (−0.061 to 0.088)	−0.033 (−0.082 to 0.015)

a Abbreviations: CI = Confidence interval; COD = People with carotid occlusive disease; HF = People with heart failure; n = number; NO_2_ = Nitrogen dioxide; PM2.5 = Particulate matter with diameter <2.5 µm; PM10 = Particulate matter with diameter <10.0 µm; VCI = People with possible vascular cognitive impairment.

b These longitudinal associations are adjusted for age, sex, educational level, smoking status, weight status, area-level socioeconomic status, population density, and global cognitive functioning at baseline.

c The air pollution data were related to the six-digit postal code areas where participants were living.

d None of the presented associations was statistically significant (i.e., p-value<0.05).

### 3.3 Mediation effects of WMH, TBV and CBF

**Fig. 3:**
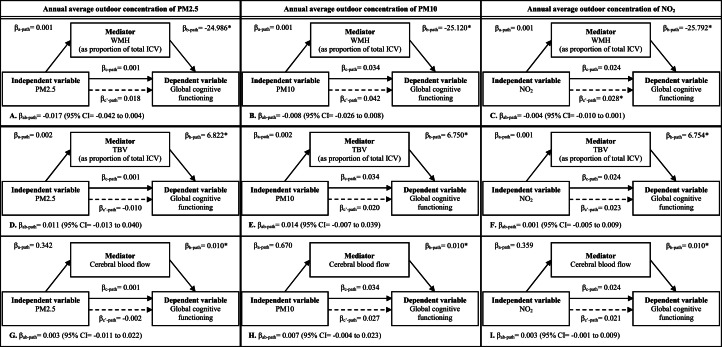
Mediation effects of white matter hyperintensities (Models A-C), total brain volume (Models D-F), and cerebral blood flow (Models G-I) in the cross-sectional associations of PM2.5, PM10 and NO_2_ with global cognitive functioning in the full sample at baseline^a,b,c^. Footnotes: ^a^Abbreviations: CI = Confidence interval; ICV = Intracranial volume; PM2.5 = Particulate matter with diameter <2.5 µm; PM10 = Particulate matter with diameter <10.0 µm; NO_2_ = nitrogen dioxide; TBV = Total brain volume; WMH = White matter hyperintensities. ^b^The c-path represents the total effect of the relevant area-level exposure measure on global cognitive functioning at baseline. The c’-path represents the direct effect of the relevant area-level exposure measure on global cognitive functioning, after adjustment for the mediator. The ab-path represents the indirect effect. The indirect effect is estimated as the multiplication of the effect of the relevant area-level exposure measure on the mediator (i.e., a-path) and the effect of the mediator on global cognitive functioning (i.e., b-path), after adjustment for the area-level exposure measure. All associations in the mediation analyses were also adjusted for all other included covariates at baseline (i.e., age, sex, educational level, smoking status, weight status, area-level socioeconomic status and population density). ^c^Level of significance: * p-value<0.05.

The mediation analyses did not indicate statistically significant mediation effects of WMH (Fig. 3, Models A-C), TBV (Fig. 3, Models D-F) and CBF (Fig. 3, Models G-I) in the cross-sectional associations of PM2.5, PM10, and NO_2_ with global cognitive functioning in the full sample. The mediation effects seemed to be missing due to the absence of associations between the exposure measures and mediators (Fig. 3). Furthermore, no mediation effects of WMH (Supplementary File 2, Figures 1.1–1.3), TBV (Supplementary File 2, Figures 2.1–2.3), and CBF (Supplementary File 2, Figures 3.1–3.3) were observed in the cross-sectional associations of air pollution with global cognitive functioning in each separate participant group.

### 3.4 Sensitivity analyses

The sensitivity analyses, in which participants from AUMC-VUmc were excluded due to missing data on relocation between baseline and two-year follow-up, did not change any of the conclusions of this study (Supplementary File 2, Table 2.1).

Air pollution was not significantly associated with any of the four cognitive domains (i.e., memory, language, attention-psychomotor speed, and executive functioning) in the full cross-sectional and longitudinal study samples (Supplementary File 2, Tables 3.1 and 3.2). Furthermore, the multi-pollutant models did not reveal statistically significant associations between any air pollutant and global cognitive functioning in the full cross-sectional (Table 4) and longitudinal (Table 5) study samples.

**Table 4:** Cross-sectional associations of air pollution with global cognitive functioning at baseline in multi-pollutant models. a^,^^b^^,^^c^^,^d.

Variables	Total (n = 341)	Reference participants (n = 89)	HF (n = 89)	COD (n = 78)	VCI (n = 85)
	β (95 % CI)	β (95 % CI)	β (95 % CI)	β (95 % CI)	β (95 % CI)
**Annual average outdoor concentration of** **PM2.5 and PM10**					
Global cognitive functioning PM2.5 PM10	−0.166 (−0.394 to 0.062) 0.163 (−0.032 to 0.359)	−0.125 (−0.361 to 0.111) 0.099 (−0.097 to 0.295)	−0.097 (−0.360 to 0.166) 0.043 (−0.204 to 0.290)	0.003 (−0.364 to 0.370) −0.050 (−0.363 to 0.262)	0.419 (−0.397 to 1.235) −0.207 (−0.906 to 0.492)
**Annual average outdoor concentration of** **PM2.5 and NO_2_**					
Global cognitive functioning PM2.5 NO_2_	−0.053 (−0.161 to 0.056) 0.030 (−0.003 to 0.060)	0.068 (−0.241 to 0.105) 0.014 (−0.025 to 0.054)	−0.059 (−0.171 to 0.054) −0.003 (−0.034 to 0.040)	−0.023 (−0.220 to 0.174) −0.012 (−0.074 to 0.049)	0.144 (−0.140 to 0.427) 0.032 (−0.048 to 0.113)
**Annual average outdoor concentration of** **PM10 and NO_2_**					
Global cognitive functioning PM10 NO_2_	−0.019 (−0.119 to 0.080) 0.026 (−0.006 to 0.058)	0.001 (−0.167 to 0.168) 0.003 (−0.043 to 0.049)	−0.045 (−0.157 to 0.066) −0.003 (−0.036 to 0.042)	−0.029 (−0.181 to 0.123) −0.011 (−0.067 to 0.045)	0.077 (−0.185 to 0.339) 0.036 (−0.051 to 0.123)
**Annual average outdoor concentration of** **PM2.5, PM10 and NO_2_**					
Global cognitive functioning PM2.5 PM10 NO_2_	−0.163 (−0.389 to 0.064) 0.111 (−0.096 to 0.318) 0.025 (−0.007 to 0.057)	−0.129 (−0.368 to 0.110) 0.085 (−0.145 to 0.315) 0.005 (−0.041 to 0.052)	−0.097 (−0.364 to 0.171) 0.041 (−0.223 to 0.306) 0.001 (−0.039 to 0.040)	0.042 (−0.369 to 0.454) −0.058 (−0.374 to 0.259) −0.014 (−0.076 to 0.049)	0.602 (−0.262 to 1.467) −0.447 (−1.242 to 0.349) 0.057 (−0.034 to 0.149)

a Abbreviations: CI = Confidence interval; COD = People with carotid occlusive disease; HF = People with heart failure; n = Number; NO_2_ = Nitrogen dioxide; PM2.5 = Particulate matter with diameter <2.5 µm; PM10 = Particulate matter with diameter <10.0 µm; VCI = People with possible vascular cognitive impairment.

b These cross-sectional associations are adjusted for age, sex, educational level, smoking status, weight status, area-level socioeconomic status and population density at baseline.

c The air pollution data were related to the six-digit postal code areas where participants were living.

d None of the presented associations was statistically significant (i.e., p-value<0.05).

**Table 5:** Longitudinal associations of air pollution at baseline with global cognitive functioning at two-year follow-up in multi-pollutant models.^a^^,^^b^^,^^c^^,^d.

Variables	Total (n = 180)	Reference participants (n = 64)	HF (n = 36)	COD (n = 43)	VCI (n = 37)
	β (95 % CI)	β (95 % CI)	β (95 % CI)	β (95 % CI)	β (95 % CI)
**Annual average outdoor concentration of** **PM2.5 and PM10**					
Global cognitive functioning PM2.5 PM10	0.138 (−0.031 to 0.307) −0.097 (−0.245 to 0.052)	−0.014 (−0.193 to 0.166) 0.026 (−0.118 to 0.171)	−0.299 (−0.704 to 0.105) 0.344 (−0.022 to 0.711)	0.571 (−0.029 to 1.113) −0.501 (−0.983 to 0.019)	−0.011 (−0.441 to 0.418) −0.040 (−0.401 to 0.321)
**Annual average outdoor concentration of** **PM2.5 and NO_2_**					
Global cognitive functioning PM2.5 NO_2_	−0.053 (−0.030 to 0.136) −0.008 (−0.034 to 0.017)	−0.062 (−0.226 to 0.102) 0.019 (−0.015 to 0.052)	0.120 (−0.049 to 0.289) −0.038 (−0.088 to 0.012)	0.056 (−0.297 to 0.409) 0.003 (−0.096 to 0.102)	−0.020 (−0.174 to 0.135) −0.031 (−0.085 to 0.024)
**Annual average outdoor concentration of** **PM10 and NO_2_**					
Global cognitive functioning PM10 NO_2_	0.021 (−0.056 to 0.099) −0.004 (−0.031 to 0.023)	−0.032 (−0.171 to 0.108) 0.014 (−0.021 to 0.049)	0.164 (−0.019 to 0.308) −0.048 (−0.095 to 0.001)	−0.095 (−0.369 to 0.179) 0.028 (−0.058 to 0.115)	−0.008 (−0.148 to 0.133) −0.032 (−0.091 to 0.027)
**Annual average outdoor concentration of** **PM2.5, PM10 and NO_2_**					
Global cognitive functioning PM2.5 PM10 NO_2_	0.137 (−0.032 to 0.307) −0.090 (−0.248 to 0.068) −0.003 (−0.030 to 0.024)	−0.058 (−0.253 to 0.138) −0.006 (−0.170 to 0.159) 0.019 (−0.019 to 0.057)	−0.269 (−0.652 to 0.114) 0.387 (−0.038 to 0.735) −0.045 (−0.091 to 0.002)	0.648 (−0.009 to 1.286) −0.530 (−1.029 to 0.030) −0.020 (−0.118 to 0.077)	−0.097 (−0.549 to 0.356) 0.075 (−0.337 to 0.487) −0.036 (−0.100 to 0.028)

a Abbreviations: CI = Confidence interval; COD = People with carotid occlusive disease; HF = People with heart failure; n = number; NO_2_ = Nitrogen dioxide; PM2.5 = Particulate matter with diameter <2.5 µm; PM10 = Particulate matter with diameter <10.0 µm; VCI = People with possible vascular cognitive impairment.

b These longitudinal associations are adjusted for age, sex, educational level, smoking status, weight status, area-level socioeconomic status, population density, and global cognitive functioning at baseline.

c The air pollution data were related to the six-digit postal code areas where participants were living.

d None of the presented associations was statistically significant (i.e., p-value<0.05).

## 4 Discussion

This study examined both cross-sectional and longitudinal associations between air pollution and global cognitive functioning in individuals without or with HF, COD, or VCI. Furthermore, this study examined whether the associations differed by participant group, and whether the cross-sectional associations were mediated by WMH, TBV, and CBF. No supportive evidence was found for either cross-sectional or longitudinal associations between air pollution and global cognitive functioning. No effect modification by participant group was found, and none of the three brain health indicators significantly mediated the cross-sectional associations of air pollution with global cognitive functioning.

This relatively small study provides an initial step toward understanding how specific air pollutants relate to cognitive functioning and its underlying biological mechanisms in both healthy and vulnerable individuals, by examining cross-sectional and longitudinal associations and assessing the mediating role of brain health indicators in reference participants and a clinical sample of individuals with cardiovascular disorders along the heart-brain axis [6,12,13,20,53,54]. A particular strength of this study is the integration of high-quality air pollution data on a detailed spatial scale, combined with MRI-based data on brain health indicators from a multi-center study. This approach allows for the assessment of the biological mechanisms that may link air pollution to cognitive functioning [6,12,13,20,53].

This study has several limitations to consider. First, the sample size was fairly small, which resulted in less precision around the estimates and, consequently, a lower ability to detect statistically significant associations. Second, due to the relatively short follow-up period and the lack of (substantial) changes in air pollution over time, we were unable to appropriately examine whether changes in air pollution concentrations were associated with changes in global cognitive functioning and brain health indicators. As a result, we were also unable to draw stronger conclusions about the causal relationship between air pollution and cognitive functioning and brain health indicators. Third, we determined exposure to air pollution in the residential six-digit postal code area of participants. Although this is a commonly used, detailed spatial level for residential environmental exposure assessment, we did not consider exposure to air pollution at other places where people spend substantial amounts of time (e.g., shopping and recreation areas). This could have led to some exposure misclassification. However, as shown by Hoek and colleagues, such misclassification is likely small [55]. Finally, although we adjusted for relevant covariates in our analyses, there might still be residual confounding factors that we did not account for, such as occupational exposure and physical activity.

Previous larger studies have shown small, but statistically significant, negative effects of air pollution on cognitive functioning in middle-aged and older adults [[9], [10], [11]]. The present study also indicates small effect estimates, which is typical for studies on the health effects of air pollution [[56], [57], [58]]. However, the observed associations were inconsistent in direction and not statistically significant. This is remarkable, because almost all participants (i.e., %_PM2.5_ = 100.0 %; %_PM10_ = 98.8 %; %_NO2_ = 100.0 %) were exposed to air pollution levels higher than the recommended annual average exposure levels in the World Health Organization 2021 Global Air Quality Guidelines [59]. A potential explanation for the lack of significant associations in this study, where most participants live in urban areas with relatively high exposure levels, could be the limited variation in exposure. Future research could include both healthy reference participants and patient groups from rural as well as urban areas, increasing exposure variability and enabling more robust assessment of potential associations.

Previous studies have shown that higher levels of air pollution are related to increased WMH and decreased TBV and CBF [11,13,19]. In the present study, we found no evidence that the association between air pollution and global cognitive functioning was mediated by any of these brain health indicators. Overall, the magnitude of the various cross-sectional and longitudinal associations was small, and the observed associations did not follow a conclusive pattern. The latter might be due to limited statistical power in this study.

Future research could replicate our approach with data from a larger number of participants and over a longer follow-up period. That would increase statistical power and enable appropriate examination of whether changes in air pollution are associated with changes in global cognitive functioning, or with changes in WMH, TBV, and CBF over time (e.g., assessed using more sensitive ultra-high-field 7T MRI scanners). The latter would provide insight into the causal relationship between air pollution, cognitive functioning, and brain health indicators. Furthermore, future research could incorporate other potentially relevant exposure measures. For example, ultrafine particulate matter with diameters <0.1 µm may be better able to cross the blood-brain barrier than PM2.5, PM10, or NO_2_, and may more strongly affect brain health indicators [60]. Future studies could also examine associations of air pollution with other potentially relevant biological intermediates of cognitive functioning, including neuro-inflammation and oxidative stress. Finally, future studies could not only consider exposure to air pollution in the residential environment, but could also use time-activity-weighted exposure measures that consider exposures at other significant places where individuals spend their time [7,55,61]. This could improve the assessment of air pollution exposure and reduce bias when estimating associations of air pollution with global cognitive functioning and brain health indicators.

In conclusion, air pollution was not associated with global cognitive functioning in healthy individuals and those with cardiovascular disorders along the heart-brain axis (i.e., HF, COD, or VCI) in this study. Furthermore, the associations between air pollution and global cognitive functioning did not differ by participant group. Additionally, the associations of PM2.5, PM10 and NO_2_ with global cognitive functioning were not significantly mediated by WMH, TBV and CBF. The results of the present study are based on relatively small study samples; therefore, these exploratory findings need to be interpreted with caution.

## Ethics approval and consent to participate

Ethical approval was obtained from the Review Board of Leiden University Medical Center (P.14.002). All participants have provided informed consent.

## Consent for publication

Not applicable.

## Availability of data and materials

The data of the Heart Brain Study are available on request, provided that an agreement is made up with the steering group of the Heart-Brain Connection Consortium.

## Funding sources

This work is part of the Heart-Brain Connection crossroads (HBCx) consortium of the Dutch CardioVascular Alliance. HBCx has received funding from the Dutch Heart Foundation under grant agreements 2018–28 and CVON 2012–06.

The geo-data were collected as part of the Geoscience and Health Cohort Consortium, which was financially supported by the Netherlands Organization for Scientific Research (NWO) – the Netherlands Organization for Health Research and Development (project number: 91118017), and the Amsterdam UMC.

EJT is supported by a NWO Gravitation Grant (Exposome-NL, 024.004.017). FJW is supported by the Netherlands Organization for Health Research and Development (BIRD-NL, project number 60-65700-98-027).

The funding bodies had no role in the study design, the collection, analysis and interpretation of the data, in writing of the manuscript and in the decision to submit the manuscript for publication.

## CRediT authorship contribution statement

**Erik J. Timmermans:** Writing – original draft, Methodology, Investigation, Formal analysis, Data curation, Conceptualization. **Esther E. Bron:** Writing – review & editing, Validation, Methodology, Investigation, Formal analysis, Data curation. **Michiel L. Bots:** Writing – review & editing, Project administration, Methodology, Investigation, Data curation, Conceptualization. **Anna E. Leeuwis:** Writing – review & editing, Methodology, Investigation, Formal analysis, Data curation. **Justine E.F. Moonen:** Writing – review & editing, Methodology, Investigation, Formal analysis, Data curation. **Frank J. Wolters:** Writing – review & editing, Methodology, Investigation, Formal analysis, Data curation. **Geert Jan Biessels:** Writing – review & editing, Supervision, Project administration, Methodology, Investigation, Funding acquisition, Formal analysis, Data curation, Conceptualization. **Ilonca Vaartjes:** Writing – review & editing, Supervision, Project administration, Methodology, Investigation, Formal analysis, Conceptualization.

## Declaration of competing interest

The authors declare the following financial interests/personal relationships which may be considered as potential competing interests:

Geert Jan Biessels reports financial support was provided by Dutch Heart Foundation. Erik Timmermans reports financial support was provided by Netherlands Organization for Scientific Research. Frank Wolters reports financial support was provided by Netherlands Organization for Scientific Research. If there are other authors, they declare that they have no known competing financial interests or personal relationships that could have appeared to influence the work reported in this paper.

## Appendix Supplementary materials

Image, application 1

Image, application 2
