# Supplementary material for: Air pollution in relation to brain health indicators and global cognitive functioning in people with cardiovascular disorders along the heart-brain axis
Source: Cereb Circ Cogn Behav. 2026 Feb 28;10:100535. doi: 10.1016/j.cccb.2026.100535 (PMC12972734; doi:10.1016/j.cccb.2026.100535)
Supplement: Supplementary file 1 [file mmc1.docx]

**Supplementary File 1**

Air pollution in relation to brain health indicators and global cognitive functioning in people with cardiovascular disorders along the heart-brain axis

Erik J. Timmermans, PhD ^1*^, Esther E. Bron, PhD ^2^, Prof. Michiel L. Bots, PhD ^1^,

Anna E. Leeuwis, PhD ^3^_,_ Justine E.F. Moonen, PhD ^3^, Frank J. Wolters, PhD ^2,4^,

Prof. Geert Jan Biessels, PhD ^5^, Ilonca Vaartjes, PhD ^1^,

on behalf of the Heart-Brain Connection Consortium

^1^ Julius Center for Health Sciences and Primary Care, University Medical Center Utrecht, Utrecht University, Utrecht, the Netherlands.

^2^ Department of Radiology & Nuclear Medicine, Erasmus MC, Rotterdam, the Netherlands.

^3^ Alzheimer Center Amsterdam, Department of Neurology, Amsterdam Neuroscience, Vrije Universiteit Amsterdam, Amsterdam UMC, Amsterdam, the Netherlands.

^4^ Department of Epidemiology, Erasmus MC, University Medical Center Rotterdam, Rotterdam, the Netherlands.

^5^ Department of Neurology, UMC Utrecht Brain Center, University Medical Center Utrecht, Utrecht, the Netherlands.

* Corresponding author: Erik J. Timmermans, PhD

Julius Center for Health Sciences and Primary Care, University Medical Center Utrecht, Utrecht University, Utrecht, the Netherlands. Internal mail no. Str6.131. P.O. Box 85500. 3508 GA Utrecht, the Netherlands. E-mail: e.j.timmermans-5@umcutrecht.nl

**Detailed information on neuropsychological tests**

*Neuropsychological tests per cognitive domain*

Memory was examined using the Visual Association Test (VAT), part A and the total immediate recall, delayed recall and recognition score of the Dutch version of Rey’s Auditory Verbal Learning Test (RAVLT) [1,2]. Language was assessed using the VAT naming and the 1-minute category fluency (animals) [1,3,4]. Attention-psychomotor speed was examined using the Trail Making Test part A (TMT-A), the forward condition of the Digit Span, the Letter Digit Substitution Test, and the Stroop Color Word Test (SCWT) card I and II [5-11]. Executive functioning was measured using the index score of Trail Making Test part B (TMT-B)/TMT-A, the backward condition of the Digit Span and the SCWT interference score calculated as card III/([card I + card II] / 2) [5,7,9]. The RAVLT recognition score, the TMT-A, the TMT-B and the SCWT scores were inverted, so higher scores corresponds with a better performance. For the included participants where TMT-B was missing (n_sample_cross_sectional_baseline_analyses_=11; 3.2%; n_sample_longitudinal_analyses_=9; 5.0%), the missing TMT-B score was replaced by single imputation of TMT-A multiplied by the group mean TMT-B/TMT-A index score [12].

**References**

1. Lindeboom J, Schmand B, Tulner L, et al. Visual association test to

detect early dementia of the Alzheimer type. *J Neurol Neurosurg Psychiatry*. 2002;73:126-33.

2. Van der Elst W, van Boxtel MP, van Breukelen GJ, et al. Rey's verbal learning test:

normative data for 1855 healthy participants aged 24-81 years and the influence of age, sex, education, and mode of presentation. *J Int Neuropsychol Soc*. 2005;11:290-302.

3. Luteijn F, Van der Ploef FAE. *Handleiding Groninger Intelligentie Test (Manual*

*Groningen Intelligence Test)*. Lisse, the Netherlands: Swets & Zeitlinger, 1983.

4. Van der Elst W, Van Boxtel MP, Van Breukelen GJ, et al. Normative data for the

Animal, Profession and Letter M Naming verbal fluency tests for Dutch speaking participants and the effects of age, education, and sex. *J Int Neuropsychol Soc*. 2006;12:80-9.

5. Hammes J. *De Stroop-Kleur Woord Test:* *Handleiding [In English: The Stroop Color-*

*Word Test: Manual]*. Amsterdam, the Netherlands: Swets & Zeitlinger, 1973.

6. Lindeboom J, Matto D. Digit series and Knox cubes as concentration tests for elderly

subjects. *Tijdschr Gerontol Geriatr*. 1994;25:63-8.

7. Reitan RM. The relation of the trail making test to organic brain damage. *J Consult*

*Psychol*. 1955;19:393-4.

8. Stroop J. Studies on interference in serial verbal reactions. *J Exp Psychol*. 1935;18:643-

62.

9. Van der Elst W, Van Boxtel MP, Van Breukelen GJ, et al. The Stroop color-word test:

influence of age, sex, and education; and normative data for a large sample across the adult age range. *Assessment*. 2006;13:62-79.

10. Van der Elst W, Van Boxtel MP, Van Breukelen GJ, et al. The Letter Digit

Substitution Test: normative data for 1,858 healthy participants aged 24-81 from the Maastricht Aging Study (MAAS): influence of age, education, and sex. *J Clin Exp Neuropsychol*. 2006;28:998-1009.

11. Wechsler D. *WAIS-III: Wechsler Adult Intelligence Scale (3^rd^ Edition). Administration*

*and Scoring Manual*. San Antionio, Texas, United States of America: Psychological Corportation/Harcourt Brace, 2017.

12. Leeuwis AE, Hooghiemstra AM, Bron EE, et al. Cerebral blood flow and cognitive

functioning in patients with disorders along the heart-brain axis. *Alzheimers Dement (N Y)*. 2020;6:e12034.
