# Supplementary material for: Air pollution in relation to brain health indicators and global cognitive functioning in people with cardiovascular disorders along the heart-brain axis
Source: Cereb Circ Cogn Behav. 2026 Feb 28;10:100535. doi: 10.1016/j.cccb.2026.100535 (PMC12972734; doi:10.1016/j.cccb.2026.100535)
Supplement: Supplementary file 2 [file mmc2.docx]

**Supplementary File 2**

Air pollution in relation to brain health indicators and global cognitive functioning in people with cardiovascular disorders along the heart-brain axis

Erik J. Timmermans, PhD ^1*^, Esther E. Bron, PhD ^2^, Prof. Michiel L. Bots, PhD ^1^,

Anna E. Leeuwis, PhD ^3^_,_ Justine E.F. Moonen, PhD ^3^, Frank J. Wolters, PhD ^2,4^,

Prof. Geert Jan Biessels, PhD ^5^, Ilonca Vaartjes, PhD ^1^,

on behalf of the Heart-Brain Connection Consortium

^1^ Julius Center for Health Sciences and Primary Care, University Medical Center Utrecht, Utrecht University, Utrecht, the Netherlands.

^2^ Department of Radiology & Nuclear Medicine, Erasmus MC, Rotterdam, the Netherlands.

^3^ Alzheimer Center Amsterdam, Department of Neurology, Amsterdam Neuroscience, Vrije Universiteit Amsterdam, Amsterdam UMC, Amsterdam, the Netherlands.

^4^ Department of Epidemiology, Erasmus MC, University Medical Center Rotterdam, Rotterdam, the Netherlands.

^5^ Department of Neurology, UMC Utrecht Brain Center, University Medical Center Utrecht, Utrecht, the Netherlands.

* Corresponding author: Erik J. Timmermans, PhD

Julius Center for Health Sciences and Primary Care, University Medical Center Utrecht, Utrecht University, Utrecht, the Netherlands. Internal mail no. Str6.131. P.O. Box 85500. 3508 GA Utrecht, the Netherlands. E-mail: e.j.timmermans-5@umcutrecht.nl

| **Outcome measure** | **Interaction terms** | **p-value** |
| --- | --- | --- |
| Global cognitive functioning | PM2.5 x HF  PM2.5 x COD  PM2.5 x VCI | 0.65  0.90  **0.01** |
| Global cognitive functioning | PM10 x HF  PM10 x COD  PM10 x VCI | 0.46  0.81  **0.02** |
| Global cognitive functioning | NO_2_ x HF  NO_2_ x COD  NO_2_ x VCI | 0.69  0.95  **0.01** |

**Table 1.1: Overview of air pollution by participant group interaction terms in the cross-sectional analyses. ^a,b^**

^a^ Abbreviations: COD = People with carotid occlusive disease; HF = People with heart failure; PM2.5 = Particulate matter with diameter <2.5µm; PM10 = Particulate matter with diameter <10.0µm; NO_2_ = Nitrogen dioxide; VCI = People with possible vascular cognitive impairment.

^b^ In bold: p-value<0.05.

| **Outcome measure** | **Interaction terms** | **p-value** |
| --- | --- | --- |
| Global cognitive functioning | PM2.5 x HF  PM2.5 x COD  PM2.5 x VCI | 0.61  0.23  0.83 |
| Global cognitive functioning | PM10 x HF  PM10 x COD  PM10 x VCI | 0.50  0.93  0.99 |
| Global cognitive functioning | NO_2_ x HF  NO_2_ x COD  NO_2_ x VCI | 0.56  0.44  0.90 |

**Table 1.2: Overview of air pollution by participant group interaction terms in the longitudinal analyses. ^a^**

^a^ Abbreviations: COD = People with carotid occlusive disease; HF = People with heart failure; PM2.5 = Particulate matter with diameter <2.5µm; PM10 = Particulate matter with diameter <10.0µm; NO_2_ = Nitrogen dioxide; VCI = People with possible vascular cognitive impairment.

**Annual average outdoor concentration of PM2.5**

**Mediator**

WMH (as proportion of total ICV)

β_a-path_= 0.001

β_b-path_= -9.447

β_c-path_= -0.021

**Independent variable**

PM2.5

**Dependent variable**

Global cognitive functioning

β_c’-path_= -0.017

**Reference participants** β_ab-path_= -0.004 (95% CI= -0.061 to 0.029)

β_c’-path_= -0.057

**Mediator**

WMH (as proportion of total ICV)

β_a-path_= 0.001

β_b-path_= 17.984

β_c-path_= -0.055

**Independent variable**

PM2.5

**Dependent variable**

Global cognitive functioning

**Heart failure**  β_ab-path_= 0.002 (95% CI= -0.013 to 0.021)

**Mediator**

WMH (as proportion of total ICV)

β_a-path_= 0.001

β_b-path_= 0.397

β_c-path_= -0.052

**Independent variable**

PM2.5

**Dependent variable**

Global cognitive functioning

β_c’-path_= -0.052

**Carotid occlusive disease**  β_ab-path_= -0.001 (95% CI= -0.017 to 0.029)

**Mediator**

WMH (as proportion of total ICV)

β_a-path_= 0.001

β_b-path_= -0.566

β_c-path_= 0.190

**Independent variable**

PM2.5

**Dependent variable**

Global cognitive functioning

β_c’-path_= 0.191

**Cognitive vascular impairment** β_ab-path_= -0.001 (95% CI= -0.061 to 0.060)

**Figure 1.1: Mediation effects of volume of white matter hyperintensities in the cross-sectional associations of air pollution with global cognitive functioning in each separate participant group at baseline. ^a-c^**

**Mediator**

WMH (as proportion of total ICV)

**Annual average outdoor concentration of PM10**

β_a-path_= 0.001

β_b-path_= -12.997

β_c-path_= 0.008

β_c’-path_= 0.014

**Independent variable**

PM10

**Dependent variable**

Global cognitive functioning

**Reference participants** β_ab-path_= -0.006 (95% CI= -0.045 to 0.025)

**Mediator**

WMH (as proportion of total ICV)

β_a-path_= 0.001

β_b-path_= 18.126

β_c-path_= -0.041

**Independent variable**

PM10

**Dependent variable**

Global cognitive functioning

β_c’-path_= -0.044

**Heart failure**  β_ab-path_= 0.003 (95% CI= -0.011 to 0.024)

**Mediator**

WMH (as proportion of total ICV)

β_a-path_= 0.001

β_b-path_= 1.229

β_c-path_= -0.048

β_c’-path_= -0.048

**Independent variable**

PM10

**Dependent variable**

Global cognitive functioning

**Carotid occlusive disease** β_ab-path_= 0.001 (95% CI= -0.022 to 0.028)

β_c’-path_= 0.134

**Mediator**

WMH (as proportion of total ICV)

β_a-path_= 0.001

β_b-path_= -0.433

β_c-path_= 0.133

**Independent variable**

PM10

**Dependent variable**

Global cognitive functioning

**Cognitive vascular impairment** β_ab-path_= -0.001 (95% CI= -0.052 to 0.051)

**Figure 1.2: Mediation effects of volume of white matter hyperintensities in the cross-sectional associations of air pollution with global cognitive functioning in each separate participant group at baseline. ^a-c^**

**Annual average outdoor concentration of NO_2_**

**Mediator**

WMH (as proportion of total ICV)

β_a-path_= 0.001

β_b-path_= -13.583

β_c-path_= 0.003

**Independent variable**

NO_2_

**Dependent variable**

Global cognitive functioning

β_c’-path_= 0.005

**Reference participants** β_ab-path_= -0.002 (95% CI= -0.013 to 0.008)

**Mediator**

WMH (as proportion of total ICV)

β_a-path_= 0.001

β_b-path_= 18.977

β_c-path_= -0.005

**Independent variable**

NO_2_

**Dependent variable**

Global cognitive functioning

β_c’-path_= -0.010

**Heart failure**  β_ab-path_= 0.005 (95% CI= -0.004 to 0.015)

**Mediator**

WMH (as proportion of total ICV)

β_a-path_= 0.001

β_b-path_= 2.474

β_c-path_= -0.018

**Independent variable**

NO_2_

**Dependent variable**

Global cognitive functioning

β_c’-path_= -0.018

**Carotid occlusive disease**  β_ab-path_= 0.001 (95% CI= -0.007 to 0.017)

**Mediator**

WMH (as proportion of total ICV)

β_a-path_= 0.001

β_b-path_= -1.811

β_c-path_= 0.049

**Independent variable**

NO_2_

**Dependent variable**

Global cognitive functioning

β_c’-path_= 0.050

**Cognitive vascular impairment** β_ab-path_= -0.001 (95% CI= -0.021 to 0.017)

**Figure 1.3: Mediation effects of volume of white matter hyperintensities in the cross-sectional associations of air pollution with global cognitive functioning in each separate participant group at baseline. ^a-c^**

Footnotes:

^a^ Abbreviations: CI = Confidence interval; ICV = Intracranial volume; PM2.5 = Particulate matter with diameter <2.5µm; PM10 = Particulate matter with diameter <10.0µm; NO_2_ = Nitrogen dioxide; WMH = White matter hyperintensities

^b^ The c-path represents the total effect of the relevant area-level exposure measure on global cognitive functioning at baseline. The c’-path represents the direct effect of the relevant area-level exposure measure on global cognitive functioning, after adjustment for the mediator. The ab-path represents the indirect effect. The indirect effect is estimated as the multiplication of the effect of the relevant area-level exposure measure on the mediator (i.e., a-path) and the effect of the mediator on global cognitive functioning (i.e., b-path), after adjustment for the area-level exposure measure. All associations in the mediation analyses were also adjusted for all other included covariates at baseline (i.e., age, sex, educational level, smoking status, weight status, area-level socioeconomic status and population density).

^c^ None of the presented associations was statistically significant (i.e., p-value<0.05).

**Annual average outdoor concentration of PM2.5**

**Mediator**

TBV (as proportion of total ICV)

β_a-path_= -0.003

β_b-path_= 3.251

β_c-path_= -0.021

**Independent variable**

PM2.5

**Dependent variable**

Global cognitive functioning

β_c’-path_= -0.013

**Reference participants** β_ab-path_= -0.008 (95% CI= -0.035 to 0.013)

**Mediator**

TBV (as proportion of total ICV)

β_a-path_= -0.002

β_b-path_= 4.448*

β_c-path_= -0.055

**Independent variable**

PM2.5

**Dependent variable**

Global cognitive functioning

β_c’-path_= -0.046

**Heart failure** β_ab-path_= -0.009 (95% CI= -0.052 to 0.022)

**Mediator**

TBV (as proportion of total ICV)

β_a-path_= 0.002

β_b-path_= 5.313*

β_c-path_= -0.052

**Independent variable**

PM2.5

**Dependent variable**

Global cognitive functioning

β_c’-path_= -0.060

**Carotid occlusive disease**  β_ab-path_= 0.008 (95% CI= -0.028 to 0.051)

**Mediator**

TBV (as proportion of total ICV)

β_a-path_= 0.007*

β_b-path_= 6.926

β_c-path_= 0.190

**Independent variable**

PM2.5

**Dependent variable**

Global cognitive functioning

β_c’-path_= 0.140

**Cognitive vascular impairment** β_ab-path_= 0.050 (95% CI= -0.022 to 0.156)

**Figure 2.1: Mediation effects of total brain volume in the cross-sectional associations of air pollution with global cognitive functioning in each separate participant group at baseline. ^a-c^**

**Mediator**

TBV (as proportion of total ICV)

**Annual average outdoor concentration of PM10**

β_a-path_= -0.001

β_b-path_= 3.294

β_c-path_= 0.008

β_c’-path_= 0.009

**Independent variable**

PM10

**Dependent variable**

Global cognitive functioning

**Reference participants** β_ab-path_= -0.001 (95% CI= -0.019 to 0.024)

β_c’-path_= -0.040

**Mediator**

TBV (as proportion of total ICV)

β_a-path_= -0.001

β_b-path_= 4.540*

β_c-path_= -0.041

**Independent variable**

PM10

**Dependent variable**

Global cognitive functioning

**Heart failure**  β_ab-path_= -0.001 (95% CI= -0.037 to 0.026)

**Mediator**

TBV (as proportion of total ICV)

β_a-path_= 0.001

β_b-path_= 5.320*

β_c-path_= -0.048

β_c’-path_= -0.055

**Independent variable**

PM10

**Dependent variable**

Global cognitive functioning

**Carotid occlusive disease** β_ab-path_= 0.007 (95% CI= -0.021 to 0.049)

**Mediator**

TBV (as proportion of total ICV)

β_a-path_= 0.004

β_b-path_= 7.391

β_c-path_= 0.133

**Independent variable**

PM10

**Dependent variable**

Global cognitive functioning

β_c’-path_= 0.105

**Cognitive vascular impairment** β_ab-path_= 0.028 (95% CI= -0.028 to 0.104)

**Figure 2.2: Mediation effects of total brain volume in the cross-sectional associations of air pollution with global cognitive functioning in each separate participant group at baseline. ^a-c^**

**Annual average outdoor concentration of NO_2_**

β_c’-path_= 0.003

**Mediator**

TBV (as proportion of total ICV)

β_a-path_= -0.001

β_b-path_= 3.299

β_c-path_= 0.003

**Independent variable**

NO_2_

**Dependent variable**

Global cognitive functioning

**Reference participant**s β_ab-path_= -0.001 (95% CI= -0.005 to 0.006)

**Mediator**

TBV (as proportion of total ICV)

β_a-path_= -0.002

β_b-path_= 4.643*

β_c-path_= -0.005

**Independent variable**

NO_2_

**Dependent variable**

Global cognitive functioning

β_c’-path_= 0.004

**Heart failure** β_ab-path_= -0.009 (95% CI= -0.023 to 0.001)

**Mediator**

TBV (as proportion of total ICV)

β_a-path_= 0.001

β_b-path_= 5.400*

β_c-path_= -0.018

β_c’-path_= -0.022

**Independent variable**

NO_2_

**Dependent variable**

Global cognitive functioning

**Carotid occlusive disease** β_ab-path_= 0.004 (95% CI= -0.009 to 0.021)

**Mediator**

TBV (as proportion of total ICV)

β_a-path_= -0.001

β_b-path_= 8.096*

β_c-path_= 0.049

**Independent variable**

NO_2_

**Dependent variable**

Global cognitive functioning

β_c’-path_= 0.051

**Cognitive vascular impairment** β_ab-path_= -0.002 (95% CI= -0.024 to 0.019)

**Figure 2.3: Mediation effects of total brain volume in the cross-sectional associations of air pollution with global cognitive functioning in each separate participant group at baseline. ^a-c^**

Footnotes:

^a^ Abbreviations: CI = Confidence interval; ICV = Intracranial volume; PM2.5 = Particulate matter with diameter <2.5µm; PM10 = Particulate matter with diameter <10.0µm; NO_2_ = Nitrogen dioxide; TBV = Total brain volume.

^b^ The c-path represents the total effect of the relevant area-level exposure measure on global cognitive functioning at baseline. The c’-path represents the direct effect of the relevant area-level exposure measure on global cognitive functioning, after adjustment for the mediator. The ab-path represents the indirect effect. The indirect effect is estimated as the multiplication of the effect of the relevant area-level exposure measure on the mediator (i.e., a-path) and the effect of the mediator on global cognitive functioning (i.e., b-path), after adjustment for the area-level exposure measure. All associations in the mediation analyses were also adjusted for all other included covariates at baseline (i.e., age, sex, educational level, smoking status, weight status, area-level socioeconomic status and population density).

^c^ Level of significance: * p-value<0.05.

**Annual average outdoor concentration of PM2.5**

**Mediator**

Cerebral blood flow

β_a-path_= -0.579

β_b-path_= 0.006

β_c-path_= -0.021

**Independent variable**

PM2.5

**Dependent variable**

Global cognitive functioning

β_c’-path_= -0.018

**Reference participants** β_ab-path_= -0.003 (95% CI= -0.026 to 0.031)

**Mediator**

Cerebral blood flow

β_a-path_= 0.355

β_b-path_= 0.001

β_c-path_= -0.055

**Independent variable**

PM2.5

**Dependent variable**

Global cognitive functioning

β_c’-path_= -0.056

**Heart failure** β_ab-path_= 0.001 (95% CI= -0.014 to 0.013)

**Mediator**

Cerebral blood flow

β_a-path_= -0.934

β_b-path_= 0.003

β_c-path_= -0.052

**Independent variable**

PM2.5

**Dependent variable**

Global cognitive functioning

β_c’-path_= -0.049

**Carotid occlusive disease** β_ab-path_= -0.003 (95% CI= -0.028 to 0.021)

**Mediator**

Cerebral blood flow

β_a-path_= 2.237

β_b-path_= 0.008

β_c-path_= 0.190

**Independent variable**

PM2.5

**Dependent variable**

Global cognitive functioning

β_c’-path_= 0.172

**Cognitive vascular impairment** β_ab-path_= 0.018 (95% CI= -0.043 to 0.105)

**Figure 3.1: Mediation effects of cerebral blood flow in the cross-sectional associations of air pollution with global cognitive functioning in each separate participant group at baseline. ^a-c^**

**Annual average outdoor concentration of PM10**

**Mediator**

Cerebral blood flow

β_a-path_= 0.864

β_b-path_= 0.006

β_c-path_= 0.008

**Independent variable**

PM10

**Dependent variable**

Global cognitive functioning

β_c’-path_= 0.003

**Reference participants** β_ab-path_= 0.005 (95% CI= -0.017 to 0.032)

**Mediator**

Cerebral blood flow

β_a-path_= 0.084

β_b-path_= 0.001

β_c-path_= -0.041

**Independent variable**

PM10

**Dependent variable**

Global cognitive functioning

β_c’-path_= -0.041

**Heart failure**  β_ab-path_= 0.001 (95% CI= -0.014 to 0.010)

**Mediator**

Cerebral blood flow

β_a-path_= -0.572

β_b-path_= 0.003

β_c-path_= -0.048

**Independent variable**

PM10

**Dependent variable**

Global cognitive functioning

β_c’-path_= -0.046

**Carotid occlusive disease** β_ab-path_= -0.002 (95% CI= -0.019 to 0.017)

**Mediator**

Cerebral blood flow

β_a-path_= 1.536

β_b-path_= 0.009

β_c-path_= 0.133

**Independent variable**

PM10

**Dependent variable**

Global cognitive functioning

β_c’-path_= 0.119

**Cognitive vascular impairment** β_ab-path_= 0.014 (95% CI= -0.033 to 0.082)

**Figure 3.2: Mediation effects of cerebral blood flow in the cross-sectional associations of air pollution with global cognitive functioning in each separate participant group at baseline. ^a-c^**

**Annual average outdoor concentration of NO_2_**

**Mediator**

Cerebral blood flow

β_a-path_= 0.239

β_b-path_= 0.006

β_c-path_= 0.003

**Independent variable**

NO_2_

**Dependent variable**

Global cognitive functioning

β_c’-path_= 0.001

**Reference participants** β_ab-path_= 0.001 (95% CI= -0.003 to 0.008)

**Mediator**

Cerebral blood flow

β_a-path_= 0.500

β_b-path_= 0.001

β_c-path_= -0.005

**Independent variable**

NO_2_

**Dependent variable**

Global cognitive functioning

β_c’-path_= -0.006

**Heart failure**  β_ab-path_= 0.001 (95% CI= -0.005 to 0.007)

**Mediator**

Cerebral blood flow

β_a-path_= -0.017

β_b-path_= 0.004

β_c-path_= -0.018

**Independent variable**

NO_2_

**Dependent variable**

Global cognitive functioning

β_c’-path_= -0.018

**Carotid occlusive disease**  β_ab-path_= 0.001 (95% CI= -0.006 to 0.008)

**Mediator**

Cerebral blood flow

β_a-path_= 0.419

β_b-path_= 0.009

β_c-path_=0.049

**Independent variable**

NO_2_

**Dependent variable**

Global cognitive functioning

β_c’-path_= 0.045

**Cognitive vascular impairment** β_ab-path_= 0.004 (95% CI= -0.012 to 0.022)

**Figure 3.3: Mediation effects of cerebral blood flow in the cross-sectional associations of air pollution with global cognitive functioning in each separate participant group at baseline. ^a-c^**

Footnotes:

^a^ Abbreviations: CI = Confidence interval; PM2.5 = Particulate matter with diameter <2.5µm; PM10 = Particulate matter with diameter <10.0µm; NO_2_ = Nitrogen dioxide.

^b^ The c-path represents the total effect of the relevant area-level exposure measure on global cognitive functioning at baseline. The c’-path represents the direct effect of the relevant area-level exposure measure on global cognitive functioning, after adjustment for the mediator. The ab-path represents the indirect effect. The indirect effect is estimated as the multiplication of the effect of the relevant area-level exposure measure on the mediator (i.e., a-path) and the effect of the mediator on global cognitive functioning (i.e., b-path), after adjustment for the area-level exposure measure. All associations in the mediation analyses were also adjusted for all other included covariates at baseline (i.e., age, sex, educational level, smoking status, weight status, area-level socioeconomic status, and population density).

^c^ None of the presented associations was statistically significant (i.e., p-value<0.05).

**Table 2.1: Longitudinal associations of air pollution at baseline with global cognitive functioning at two-year follow-up up [sensitivity analyses in which participants from Amsterdam UMC, location VU University medical center, were excluded]. ^a-d^**

| **Variables** | **Total**  **(n=102)** | **Reference participants**  **(n=46)** | **HF**  **(n=7)** | **COD**  **(n=43)** | **VCI**  **(n=6)** |
| --- | --- | --- | --- | --- | --- |
|  | β (95% CI) | β (95% CI) | β (95% CI) | β (95% CI) | β (95% CI) |
| **Annual average outdoor concentration of PM2.5** |  |  |  |  |  |
| Global cognitive functioning | 0.080 (-0.054 to 0.213) | -0.065 (-0.204 to 0.074) | - ^e^ | 0.064 (-0.202 to 0.329) | - ^e^ |
|  |  |  |  |  |  |
| **Annual average outdoor concentration of PM10** |  |  |  |  |  |
| Global cognitive functioning | 0.003 (-0.114 to 0.119) | -0.017 (-0.132 to 0.098) | - ^e^ | -0.051 (-0.286 to 0.185) | - ^e^ |
|  |  |  |  |  |  |
| **Annual average outdoor concentration of NO_2_** |  |  |  |  |  |
| Global cognitive functioning | 0.006 (-0.029 to 0.041) | -0.003 (-0.029 to 0.024) | - ^e^ | 0.013 (-0.061 to 0.088) | - ^e^ |

Footnotes:

^a^ Abbreviations: CI = Confidence interval; COD = People with carotid occlusive disease; HF = People with heart failure; n = Number; NO_2_ = Nitrogen dioxide; PM2.5 = Particulate matter with diameter <2.5µm; PM10 = Particulate matter with diameter <10.0µm; VCI = People with possible vascular cognitive impairment.

^b^ These longitudinal associations are adjusted for age, sex, educational level, smoking status, weight status, area-level socioeconomic status, population density, and global cognitive functioning at baseline.

^c^ The air pollution data were related to the six-digit postal code areas where participants were living.

^d^ None of the presented associations was statistically significant (i.e., p-value<0.05).

^e^ This model could not be fitted because the number of observations was less than the number of model parameters.

**Table 3.1: Cross-sectional associations of air pollution with multiple domains of cognitive functioning at baseline. ^a-d^**

| **Variables** | **Total**  **(n=341)** | **Reference participants**  **(n=89)** | **HF**  **(n=89)** | **COD**  **(n=78)** | **VCI**  **(n=85)** |
| --- | --- | --- | --- | --- | --- |
|  | β (95% CI) | β (95% CI) | β (95% CI) | β (95% CI) | β (95% CI) |
| **Annual average outdoor concentration of PM2.5** |  |  |  |  |  |
| Memory | 0.016 (-0.184 to 0.216) | -0.062 (-0.027 to 0.149) | -0.047 (-0.268 to 0.174) | -0.106 (-0.393 to 0.180) | 0.357 (-0.274 to 0.987) |
| Language | -0.022 (-0.103 to 0.059) | 0.047 (-0.089 to 0.182) | -0.065 (-0.159 to 0.028) | -0.038 (-0.160 to 0.83) | 0.034 (-0.199 to 0.267) |
| Attention-psychomotor speed | 0.077 (-0.037 to 0.192) | -0.002 (-0.176 to 0.171) | 0.048 (-0.108 to 0.205) | -0.093 (-0.307 to 0.122) | 0.324 (-0.013 to 0.635) |
| Executive functioning | -0.034 (-0.126 to 0.057) | -0.058 (-0.233 to 0.118) | -0.162 (-0.307 to 0.016) | 0.030 (-0.150 to 0.210) | -0.069 (-0.172 to 0.309) |
|  |  |  |  |  |  |
| **Annual average outdoor concentration of PM10** |  |  |  |  |  |
| Memory | 0.063 (-0.109 to 0.236) | 0.029 (-0.145 to 0.203) | 0.003 (-0.204 to 0.211) | -0.096 (-0.340 to 0.148) | 0.262 (-0.280 to 0.803) |
| Language | -0.011 (-0.080 to 0.059) | 0.033 (-0.079 to 0.146) | -0.065 (-0.154 to 0.024) | -0.049 (-0.153 to 0.054) | -0.010 (-0.210 to 0.190) |
| Attention-psychomotor speed | -0.089 (-0.009 to 0.187) | 0.028 (-0.116 to 0.171) | 0.055 (-0.092 to 0.202) | -0.072 (-0.255 to 0.111) | 0.250 (-0.013 to 0.514) |
| Executive functioning | -0.013 (-0.092 to 0.066) | -0.034 (-0.180 to 0.111) | **-0.167 (-0.303 to -0.031)** | 0.026 (-0.128 to 0.179) | 0.037 (-0.169 to 0.244) |
|  |  |  |  |  |  |
| **Annual average outdoor concentration of NO_2_** |  |  |  |  |  |
| Memory | 0.025 (-0.030 to 0.081) | 0.009 (-0.039 to 0.056) | -0.004 (-0.077 to 0.068) | **-**0.042 (-0.132 to 0.048) | 0.077 (-0.103 to 0.257) |
| Language | 0.014 (-0.008 to 0.037) | 0.014 (-0.017 to 0.045) | -0.015 (-0.046 to 0.016) | -0.010 (-0.048 to 0.028) | 0.009 (-0.057 to 0.075) |
| Attention-psychomotor speed | 0.038 (-0.006 to 0.038) | 0.007 (-0.032 to 0.047) | 0.011 (-0.040 to 0.062) | -0.014 (-0.082 to 0.054) | 0.085 (-0.001 to 0.171) |
| Executive functioning | 0.013 (-0.012 to 0.039) | -0.016 (-0.056 to 0.024) | -0.016 (-0.065 to 0.034) | -0.004 (-0.061 to 0.053) | 0.025 (-0.043 to 0.093) |

Footnotes:

^a^ Abbreviations: CI = Confidence interval; COD = People with carotid occlusive disease; HF = people with heart failure; n = Number; NO_2_ = Nitrogen dioxide; PM2.5 = Particulate matter with diameter <2.5µm; PM10 = Particulate matter with diameter <10.0µm; VCI = People with possible vascular cognitive impairment.

^b^ These cross-sectional associations are adjusted for age, sex, educational level, smoking status, weight status, area-level socioeconomic status and population density at baseline.

^c^ The air pollution data were related to the six-digit postal code areas where participants were living.

^d^ In bold: p-value<0.05.

**Table 3.2: Longitudinal associations of air pollution at baseline with multiple domains of cognitive functioning at two-year follow-up. ^a-d^**

| **Variables** | **Total**  **(n=180)** | **Reference participants**  **(n=64)** | **HF**  **(n=36)** | **COD**  **(n=43)** | **VCI**  **(n=37)** |
| --- | --- | --- | --- | --- | --- |
|  | β (95% CI) | β (95% CI) | β (95% CI) | β (95% CI) | β (95% CI) |
| **Annual average outdoor concentration of PM2.5** |  |  |  |  |  |
| Memory | 0.106 (-0.061 to 0.274) | 0.138 (-0.063 to 0.340) | 0.107 (-0.215 to 0.429) | 0.178 (-0.501 to 0.858) | -0.265 (-0.557 to 0.027) |
| Language | 0.054 (-0.013 to 0.122) | 0.004 (-0.145 to 0.153) | 0.012 (-0.150 to 0.173) | -0.024 (-0.196 to 0.147) | 0.165 (-0.073 to 0.403) |
| Attention-psychomotor speed | -0.037 (-0.161 to 0.086) | -0.093 (-0.243 to 0.056) | -0.068 (-0.324 to 0.189) | 0.066 (-0.432 to 0.565) | -0.079 (-0.281 to 0.123) |
| Executive functioning | -0.043 (-0.127 to 0.040) | 0.064 (-0.102 to 0.229) | -0.125 (-0.431 to 0.180) | -0.063 (-0.268 to 0.142) | -0.025 (-0.195 to 0.145) |
|  |  |  |  |  |  |
| **Annual average outdoor concentration of PM10** |  |  |  |  |  |
| Memory | 0.043 (-0.104 to 0.191) | 0.110 (-0.052 to 0.271) | 0.166 (-0.118 to 0.451) | -0.112 (-0.713 to 0.490) | -0.230 (-0.473 to 0.014) |
| Language | 0.063 (-0.003 to 0.122) | -0.029 (-0.145 to 0.087) | 0.007 (-0.141 to 0.155) | 0.011 (-0.145 to 0.166) | 0.183 (-0.010 to 0.376) |
| Attention-psychomotor speed | -0.080 (-0.188 to 0.028) | -0.015 (-0.136 to 0.106) | -0.065 (-0.295 to 0.164) | -0.128 (-0.569 to 0.313) | -0.088 (-0.257 to 0.729) |
| Executive functioning | -0.016 (-0.090 to 0.057) | 0.051 (-0.078 to 0.180) | 0.002 (-0.273 to 0.277) | -0.043 (-0.222 to 0.134) | -0.010 (-0.134 to 0.114) |
|  |  |  |  |  |  |
| **Annual average outdoor concentration of NO_2_** |  |  |  |  |  |
| Memory | 0.034 (-0.018 to 0.085) | 0.033 (-0.007 to 0.073) | -0.013 (-0.111 to 0.085) | 0.062 (-0.130 to 0.253) | -0.029 (-0.139 to 0.082) |
| Language | -0.002 (-0.023 to 0.019) | 0.005 (-0.025 to 0.035) | **-0.052 (-0.090 to -0.014)** | 0.002 (-0.047 to 0.050) | -0.009 (-0.093 to 0.075) |
| Attention-psychomotor speed | -0.009 (-0.047 to 0.029) | -0.002 (-0.032 to 0.028) | -0.010 (-0.086 to 0.066) | -0.010 (-0.150 to 0.130) | -0.056 (-0.124 to 0.012) |
| Executive functioning | -0.012 (-0.038 to 0.013) | 0.005 (-0.028 to 0.039) | **-0.083 (-0.165 to -0.001)** | -0.030 (-0.088 to 0.029) | -0.042 (-0.098 to 0.014) |

Footnotes:

^a^ Abbreviations: CI = Confidence interval; COD = People with carotid occlusive disease; HF = People with heart failure; n = Number; NO_2_ = Nitrogen dioxide; PM2.5 = Particulate matter with diameter <2.5µm; PM10 = Particulate matter with diameter <10.0µm; VCI = People with possible vascular cognitive impairment.

^b^ These longitudinal associations are adjusted for age, sex, educational level, smoking status, weight status, area-level socioeconomic status, population density, and the relevant cognitive functioning outcome measure at baseline.

^c^ The air pollution data were related to the six-digit postal code areas where participants were living.

^d^ In bold: p-value<0.05.
